# Inappropriate sinus tachycardia in post-COVID-19 syndrome

**DOI:** 10.1038/s41598-021-03831-6

**Published:** 2022-01-07

**Authors:** Júlia Aranyó, Victor Bazan, Gemma Lladós, Maria Jesús Dominguez, Felipe Bisbal, Marta Massanella, Axel Sarrias, Raquel Adeliño, Ariadna Riverola, Roger Paredes, Bonaventura Clotet, Antoni Bayés-Genís, Lourdes Mateu, Roger Villuendas

**Affiliations:** 1 Cardiology Department, Heart institute, Hospital Universitari Germans Trias I Pujol, Carretera de Canyet s/n, 08916 Badalona, Spain; 2 Department of Infectious Disease, Hospital Universitari Germans Trias I Pujol, Badalona, Spain; 3 Emergency Department, Hospital Universitari Germans Trias I Pujol, Badalona, Spain; 4 AIDS Research Institute (IrsiCaixa), Badalona, Spain; 5 Centro de Investigación Biomédica en Red Enfermedades Cardiovascualres (CIBERCV), Madrid, Spain; 6 Autonomous University of Barcelona, Barcelona, Spain; 7 Centro de Investigación Biomédica en Red Enfermedades Respiratorias (CIBERES), Madrid, Spain

**Keywords:** Cardiology, Cardiovascular biology, Cardiovascular diseases

## Abstract

Inappropriate sinus tachycardia (IST) is a common observation in patients with post-COVID-19 syndrome (PCS) but has not yet been fully described to date. To investigate the prevalence and the mechanisms underlying IST in a prospective population of PCS patients. Consecutive patients admitted to the PCS Unit between June and December 2020 with a resting sinus rhythm rate ≥ 100 bpm were prospectively enrolled in this study and further examined by an orthostatic test, 2D echocardiography, 24-h ECG monitoring (heart rate variability was a surrogate for cardiac autonomic activity), quality-of-life and exercise capacity testing, and blood sampling. To assess cardiac autonomic function, a 2:1:1 comparative sub-analysis was conducted against both fully recovered patients with previous SARS-CoV-2 infection and individuals without prior SARS-CoV-2 infection. Among 200 PCS patients, 40 (20%) fulfilled the diagnostic criteria for IST (average age of 40.1 ± 10 years, 85% women, 83% mild COVID-19). No underlying structural heart disease, pro-inflammatory state, myocyte injury, or hypoxia were identified. IST was accompanied by a decrease in most heart rate variability parameters, especially those related to cardiovagal tone: pNN50 (cases 3.2 ± 3 vs. recovered 10.5 ± 8 vs. non-infected 17.3 ± 10; *p* < 0.001) and HF band (246 ± 179 vs. 463 ± 295 vs. 1048 ± 570, respectively; *p* < 0.001). IST is prevalent condition among PCS patients. Cardiac autonomic nervous system imbalance with decreased parasympathetic activity may explain this phenomenon.

## Introduction

More than 100 million people have been infected with SARS-CoV-2 worldwide. Most of these patients experience mild symptoms that do not warrant hospital admission. However, approximately 20–40% of patients remain symptomatic weeks, or even months, after overcoming the acute infection phase^1^. This phenomenon is regarded as ongoing symptomatic COVID-19 or post-COVID-19 syndrome (PCS) when remnant symptoms persist from 4 to 12 weeks and for more than 12 weeks, respectively^2^. Clinical manifestations of PCS usually include fatigue, chest pain, joint/muscle pain, dizziness, fever, shortness of breath, gastrointestinal symptoms, headache, sore throat, neurocognitive disorder, and altered sleep structure. Limited understanding of the pathological mechanisms underlying PCS represents a critical challenge to effectively testing and treating this syndrome. Injury to the autonomic nervous system (ANS) has recently been suggested to be responsible for many of the aforementioned manifestations and may be key in the pathogenesis of PCS^3^.

At the cardiovascular level, ANS dysfunction produces orthostatic syndromes, such as orthostatic hypotension and postural orthostatic tachycardia syndrome (POTS), chest pain, and cardiac arrhythmias, including inappropriate sinus tachycardia (IST)^4^. Postural orthostatic tachycardia has already been described in the setting of PCS^3,5^. In our initial experience with PCS patients, IST, which often overlaps with POTS, is also a common observation that has not been fully described to date. Similar to POTS, decreased parasympathetic activity has been postulated in the etio-pathogenesis of IST^6,7^. The aim of this study was to investigate the prevalence and underlying pathophysiological mechanisms of IST in a consecutive and prospective population of PCS patients.

## Methods

### Study population

At our institution, patients with persistent symptoms, such as tiredness, shortness of breath, dizziness, brain fog, chest pain, or headache, 3 months after an acute SARS-CoV-2 infection are referred to a multi-disciplinary PCS unit supported by infectologists, cardiologists, neurologists, rheumatologists, nutritionists, rehabilitators, and psychologists. All consecutive patients seen at this unit from June to December 2020 underwent a resting 12-lead ECG. Patients with sinus rhythm rates ≥ 100 bpm were prospectively enrolled in the study database and underwent further cardiovascular assessment. The assessment included an orthostatic test during a 10-min period of standing (to detect concomitant POTS), 2-D echocardiography, 24-h Holter monitoring, a quality-of-life test (EQ-5D-5L), 6-min walking test (6MWT), and blood sample collection to the search for biological markers of inflammation and myocardial damage. The EQ-5D-5L has five response levels: no problems (level 1), slight, moderate, severe, and extreme problems (level 5).

Secondary causes of tachycardia, such as anemia, thyroid pathology, pregnancy, infection, or pulmonary embolism, were investigated, and patients with a systemic condition justifying tachycardia were excluded from the study analysis. Patients using sympathomimetic drugs were also excluded.

### Study approval

The study was approved by the institutional ethics committee (Hospital Universitari Germans Trias i Pujol, Badalona, Barcelona, Spain; PI 20-288). The participants signed a written informed consent form before enrolling in the study. All research activities were carried out in accordance with the Declaration of Helsinki.

### Definition of inappropriate sinus tachycardia

Following conventional criteria, IST was defined as a symptomatic sinus rhythm rate ≥ 100 bpm at rest with a mean 24-h heart rate above 90 beats/min in the absence of any acute physiological demand or conditions known to commonly produce sinus tachycardia^8^.

### Assessment of cardiac autonomic function

In this study, we based our assessment of ANS imbalance on the time-and-frequency-domain heart rate variability (HRV) parameters obtained during 24-h ECG monitoring. Time-domain measurements included the average RR interval (in ms), the standard deviation of the inter-beat interval (SDNN, in ms), and the percentage of adjacent NN intervals that differed from each other by more than 50 ms (PNN50, %). Frequency-domain parameters included the very low frequency (VLF; 0.003–0.04 Hz), low frequency (LF; 0.04–0.15 Hz), and high frequency (HF; 0.15–0.40 Hz) bands. The ratio between the LF and HF bands was also calculated. Briefly, the HF and PNN50 are regarded as specific indicators of the parasympathetic influence on the heart rate, whereas the LF and VLF components have a complex physiology that integrates both the sympathetic and parasympathetic components^9^. The remaining parameters are less specific to a determined sympathetic versus parasympathetic influence on the heart rate and, thus, become less useful in characterizing a specific ANS disturbance.

In the absence of reliable reference values for the HRV parameters in the literature, we conducted a 2:1:1 comparative sub-study using two healthy populations. The sub-study included the following groups: group 1, all IST patients (cases); group 2, age- and gender-matched PCR-confirmed SARS-COV-2 patients without IST criteria; and group 3, age- and gender-matched patients who had no history of SARS-COV-2 disease, as confirmed by negative serology. Patients in group 2 were also matched by disease chronology, and their acute infection had to have the same severity and be within the same 1-month period as the corresponding cases. The severity of the infection was determined by the following criteria. Mild disease was defined as the presence of symptoms without evidence of viral pneumonia or hypoxia; moderate disease as hospitalization due to abnormal chest X-ray, hypoxia, or sepsis; and critical disease as requiring intensive care management. This 2:1:1 comparative design allowed us to establish study reference values for the assessment of HRV and to characterize presumable damage to the sympathetic versus parasympathetic input to the heart rate in the setting of PCS.

All of the Holter recordings were analyzed using an AFT 1000 + B recorder (Holter Supplies SAS, Paris, France).

### Statistical analysis

Demographic data were summarized by basic descriptive statistics in the three groups. All analyses treated the three groups independently, whereas the matching process for every two “cases” was individual. For qualitative variables, numbers and percentages within specified groups were calculated, and *p* values were obtained using χ^2^ tests. For quantitative variables, the arithmetic mean and standard deviation (SD) or median and interquartile range were reported as appropriate. Continuous variables were tested for normal distribution using Q–Q plots. The quantitative variables were compared between the three groups using a one-way ANOVA model and *p*-values for post-hoc comparisons were adjusted using the Scheffe method. All statistical analyses were performed using SPSS version 25.0 (IBM, Armonk, NY, USA). Significance was set at *p* < 0.05.

## Results

During the study period, 200 patients visited the PCS unit due to persistent symptoms beyond the third month of acute infection. Forty-four patients with a resting heart rate ≥ 100 bpm were initially screened, 4 of whom were excluded due to mean 24-h heart rate < 90 bpm (n = 2), hyperthyroidism (n = 1), or severe mitral regurgitation (n = 1). Therefore, 40 patients fulfilled the strict diagnostic criteria for IST, yielding an estimated prevalence of the disorder of 20%*.* No patient had complained of palpitations prior to the SARS-CoV-2 infection, endorsing the principle of post-infective IST. No patient was under any cardiovascular treatment at the time of the evaluation. The IST subjects had a mean heart rate of 105 ± 2 bpm supine and 125 ± 11 bpm in the upright position. The baseline characteristics of the 40 IST cases and their matched controls are presented in Table 1. All patients were Caucasian. Thirty-four (85%) were women, with a mean age of 40.1 ± 10 years. All patients had normal 2-D echocardiography results, and no remnant respiratory disease was identified in any patient. All patients had O_2_ saturation > 97%. Notably, IST patients had a higher prevalence of environmental allergy compared to the control group (25% vs. 0%; *p* = 0.01).

**Table 1:** Demographic and clinical characteristics of the cases and their matched controls.

	Inappropriate sinus tachycardia (Group 1) N = 40	Fully recovered (Group 2) N = 19	Uninfected (Group 3) N = 17	*P* value
**Demographic and clinical characteristics**				
Age (years)	40.1 ± 10	42.2 ± 11	39.5 ± 13	0.243
Males, n (%)	6 (15)	2 (11)	2 (12)	0.655
Body mass index, mean ± SD	25.2 ± 6.1	24.5 ± 3.6	22.5 ± 2.3	0.374
Smoking, n (%)	1 (3)	1(5)	0	0.623
Hypertension, n (%)	3(8)	0	0	0.589
Hyperlipidemia, n (%)	3 (8)	0	0	0.589
Diabetes mellitus, n (%)	0	0	0	NA
Asthma, n (%)	4 (10)	1 (5)	0	0.734
Environmental allergy, n (%)	10 (25)	0	0	**0.01**
**Symptoms of the acute infection**				
Palpitations, n (%)	36 (90)	1 (5)	–	**< 0.001**
Dyspnea, n (%)	33 (83)	3 (16)	–	**< 0.001**
Myalgia and joint pain, n (%)	32 (80)	17 (89)	–	0.545
Chest pain, n (%)	31 (78)	4 (21)	–	**< 0.001**
Fever, n (%)	29 (73)	16 (84)	–	0.462
Headache, n (%)	29 (73)	7 (37)	–	**0.007**
Dizziness, n (%)	21 (53)	1 (5)	–	**0.002**
Diarrhea, n (%)	21 (53)	3 (16)	–	**0.003**
Anosmia, n (%)	19 (48)	15 (79)	–	**0.03**
Ageusia, n (%)	19 (48)	8 (42)	–	0.454
Dermatologic alterations, n (%)	14 (35)	1 (5)	–	**0.009**
**Severity of clinical presentation**				
Mild	33 (83)	16 (84)	–	0.387
Moderate	6 (15)	3 (16)	–	0.550
Intensive care management	1 (3)	0	–	0.254

Values are expressed as mean ± standard deviation unless otherwise stated.

A P value of < 0.05 is considered statistically significant.

Significant values are in [bold].

During the acute phase of SARS-CoV-2 infection, 33 patients (83%) had experienced mild symptoms not requiring hospital admission; 6 patients (15%) had moderate disease with pulmonary infiltrates and required hospitalization; and only 1 patient (3%) required intensive care management. Compared to fully recovered patients, patients with PCS and IST more frequently complained of palpitations (90% vs. 5%; *p* < 0*.*001*)*, dyspnea (82% vs. 16%; *p* < 0.001), chest pain (78% vs. 21%; *p* < 0.001), headache (73% vs. 37%; *p* = 0.007), dizziness (53% vs. 5%; *p* = 0.002), diarrhea (53% vs. 16%; *p* = 0.003), and dermatological alterations (35% vs. 5%; *p* = 0.009). Interestingly, IST patients had a lower incidence of anosmia (48% vs. 79%; *p* = 0.03).

The results of the exercise capacity and quality of life assessment are presented in Table 2, along with the results of the laboratory tests. Echocardiography yielded normal results in all patients. Overall, biochemistry data were consistent with a lack of inflammation or myocardial damage at this stage after the acute phase of SARS-CoV-2 infection. The 6MWT showed that IST patients had a significantly diminished exercise capacity, with a median walking distance of 392 ± 83 m, which is only 60% of the estimated reference distance after adjusting for age, sex, and body mass index. Impaired quality of life was also identified, as suggested by a mean score in the health-state scale of 39 out of 100 points. The most affected domains were mobility (mean score 3.6), usual activities (mean score 3.5), and pain/discomfort (mean score 3).

**Table 2:** Characterization of patients with IST.

	Reference values	Mean values
**Laboratory tests**		
NT-proBNP (pg/mL)	< 125	67.6 ± 59.6
Hs-Troponin I (pg/mL)	< 14	3.09 ± 4.2
Leucocytes (× 10^9^/L)	4.00–11.00	6.83 ± 1.7
Hemoglobin (g/dL)	12.0–16.0	13.4 ± 0.7
TSH (µm/U)	0.350–4.940	1.4 ± 0.5
IL-6 (pg/mL)	0–6.40	2.2 ± 1.1
Ferritin (ng/mL)	15–160	50.4 ± 37.4
C-reactive protein (mg/L)	0.00–5.00	1.1 ± 1.3
D-Dimer (ng/mL)	0–500	247.7 ± 133.2
Fibrinogen	150–450	374.9 ± 54.1
24-h urine 3-Metoxiadrenaline (mg/L)	0.02–0.350	0.123 ± 0.120
24-h urine 3-Metoxinoradrenaline (mg/L)	0.03–0.440	0.178 ± 0.189
**Exercise capacity test**		
6MWT (m)		392.7 ± 83.2
6MWT theoretical (%)		60.1 ± 12.1

Quality of life test (EQ-5D-5L)	Score reference values^a^	Mean values
Mobility	1–5	3.6 ± 1.1
Self-care	1–5	2.4 ± 1.2
Usual activities	1–5	3.5 ± 0.9
Pain/discomfort	1–5	3.0 ± 1.0
Anxiety/depression	1–5	2.9 ± 1.2
Health state scale (Visual Analogue Scale)	0–100	39.6 ± 20.7

Values are expressed as mean ± standard deviation unless otherwise stated.

*NT-proBNP* N-terminal pro B-type natriuretic peptide, *TSH* thyroid-stimulating hormone, *IL-6* interleukin-6, *6MWT* 6-min walking test, *EQ-5D 5-L* EuroQol 5-Dimension 5-Level.

^a^Score values for each health dimension: 1: No problems, 2: Slight problems, 3: Moderate problems, 4: Severe problems, 5: Unable/Extreme. For Health state scale, 0 means the best health you can imagine, 100 means the worst health you can imagine.

**Figure 1:**
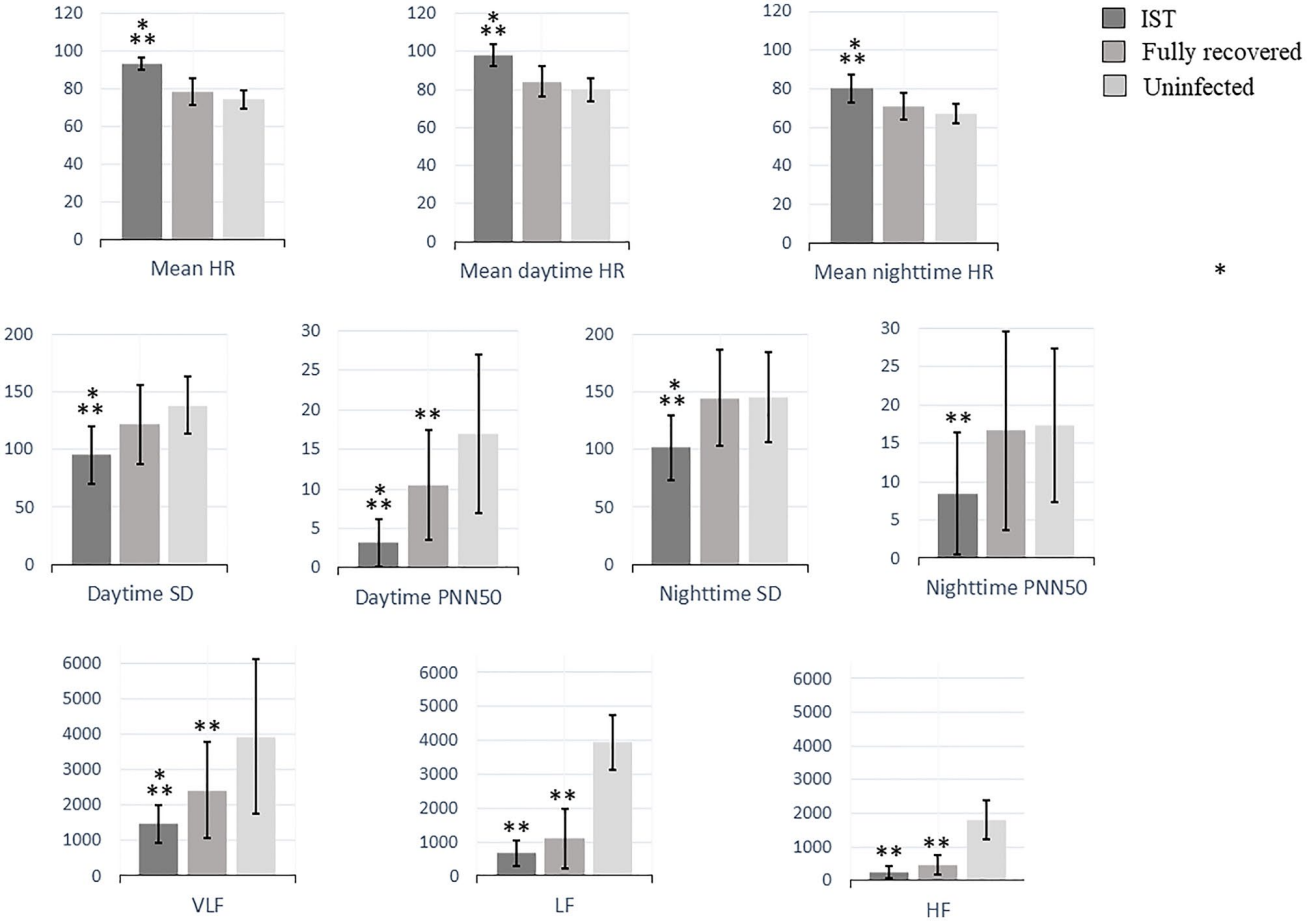
24-h ECG monitoring and HRV parameters. HRV parameters in the three studied groups: IST, fully recovered and uninfected subjects. HR indicates heart rate; PNN50, percentage of adjacent NN intervals that differ from each other by more than 50 ms; SD, standard deviation of the interbeat interval; VLF, very low frequency; LF, low frequency; HF, high frequency. A *P* value of < 0.05 is considered statistically significant. *Significant differences compared with fully recovered patients. **Significant differences compared with uninfected patients.

Results of the 24-h ECG monitoring are summarized in Table 3 and Fig. 1. Patients with IST had a higher mean heart rate, predominantly during the daytime, compared to recovered asymptomatic and uninfected subjects (98 ± 6 vs. 84 ± 8 vs. 81 ± 6 bpm, respectively; *p* < 0.001). The burden of supraventricular premature beats was lower in IST-PCS patients. No differences were observed in the maximum and minimum heart rates. All HRV variables were significantly diminished among patients with IST compared to both the recovered subjects and the uninfected group, with a significant decrease in the following time-domain parameters: daytime pNN50 (3.2 ± 3 vs. 10.5 ± 8 vs. 17.3 ± 10.0, respectively; *p* < 0.001) and daytime SDNN (95.0 ± 25 vs. 121.5 ± 34 vs. 138.1 ± 25, respectively; *p* < 0.001). A significant decrease in frequency-domain parameters was also observed in PCS patients with IST: VLF (1463.1 ± 538 vs. 2415.7 ± 1361 vs. 3931 ± 2194, respectively; *p* < 0.001), LF (670.2 ± 380 vs. 1093.2 ± 878 vs. 1801.5 ± 800, respectively; *p* < 0.001), and HF (246.0 ± 179 vs. 463.7 ± 295 vs. 1048.5 ± 570, respectively; *p* < 0.001). Numerical but non-significant differences were also observed between both control groups, with the fully recovered patients presenting with higher heart rates and lower HRV than the uninfected subjects.

**Table 3:** 24-h ECG monitoring and HRV parameters.

	Inappropriate sinus tachycardia N = 40	Fully recovered N = 19	Uninfected N = 17	*P* value		
				IST vs recovered	IST vs uninfected	Recovered vs uninfected
**Time-domain parameters**						
Mean HR (bpm)	93.6 ± 3	78.7 ± 7	74.3 ± 5	**< 0.001**	**< 0.001**	0.309
Maximum HR (bpm)	154.6 ± 16	148.9 ± 24	140.8 ± 18	0.653	0.167	0.657
Minimum HR (bpm)	59 ± 10	53.8 ± 9	51.9 ± 4	0.373	0.177	0.845
Supraventricular PB	191.6 ± 313	546.6 ± 665	955.8 ± 1180	0.172	**0.003**	0.234
Ventricular PB	85.6 ± 120	354.1 ± 542	186.9 ± 287	**0.018**	0.628	0.362
Mean daytime HR (bpm)	97.5 ± 6	84.1 ± 8	80.6 ± 6	**< 0.001**	**< 0.001**	0.656
Daytime PNN50 (%)	3.2 ± 3	10.5 ± 8	17.3 ± 10	**0.001**	**< 0.001**	**0.019**
Daytime SD (ms)	95.0 ± 25	121.5 ± 34	138.1 ± 25	**0.011**	**< 0.001**	0.270
Mean nighttime HR (bpm)	80.0 ± 7	71.2 ± 7	67.4 ± 5	**0.001**	**< 0.001**	0.405
Nighttime PNN50 (%)	8.4 ± 8	16.6 ± 15	21.4 ± 11	0.051	**0.004**	0.498
Nighttime SD (ms)	101.3 ± 28	144.5 ± 42	145.4 ± 39	**< 0.001**	**0.003**	0.997
**Spectral-domain parameters**						
VLF (Hz)	1463.1 ± 538	2415.7 ± 1361	3931.1 ± 2194	**0.044**	**< 0.001**	**0.007**
LF (Hz)	670.2 ± 380	1093.2 ± 878	1801.5 ± 800	0.092	**< 0.001**	**0.015**
HF (Hz)	246.0 ± 179	463.7 ± 295	1048.5 ± 570	0.060	**< 0.001**	**< 0.001**
LF/HF ratio (Hz)	3.6 ± 1	2.7 ± 1.3	2.0 ± 1	0.259	**0.040**	0.612

Values are presented in mean ± standard deviation.

*HR* heart rate, *SD* standard deviation of the interbeat interval, *PNN50* percentage of adjacent NN intervals that differ from each other by more than 50 ms, *VLF* very low frequency, *LF* low frequency, *HF* high frequency.

Significant values are in [bold].

**Figure 2:**
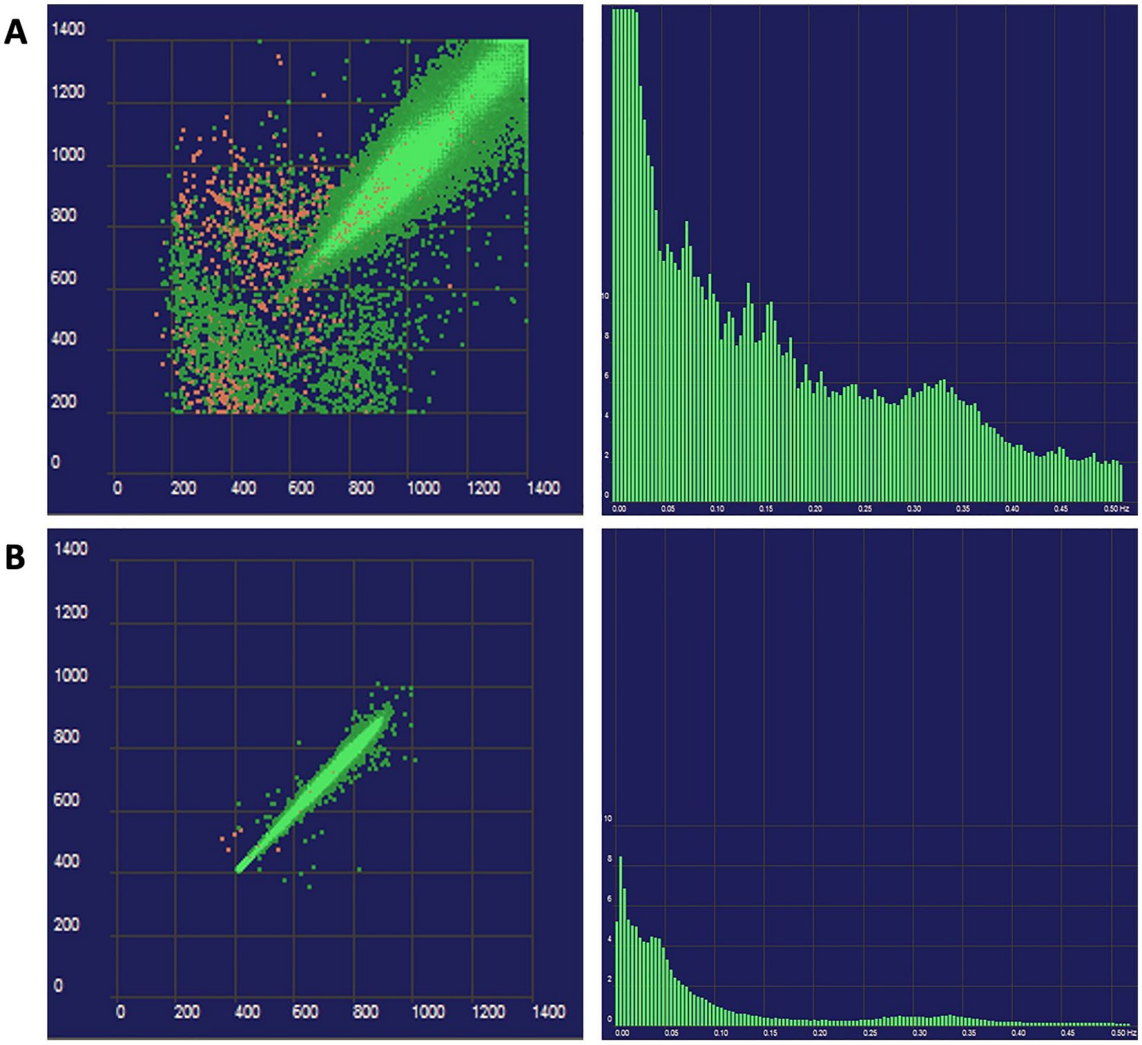
(**A**) Uninfected subject. Poincaré plot of 24-hour ECG monitoring showing the beat-to-beat variability from an uninfected subject and histogram of the frequencydomain parameters. (**B**) IST patient. Poincaré plot of 24-h ECG monitoring and histogram of the frequency-domain parameters from a patient with IST. A lower heart rate variability in comparison with the uninfected subject and an overall decrease is observed throughout all bands, being more manifest at the high frequency band (HF, 0.15–0.40 Hz), are both apparent.

An illustrative example of 24-h ECG monitoring showing altered versus normal HRV in a PCS patient vs. control is shown in Fig. 2.

## Discussion

**Figure 3:**
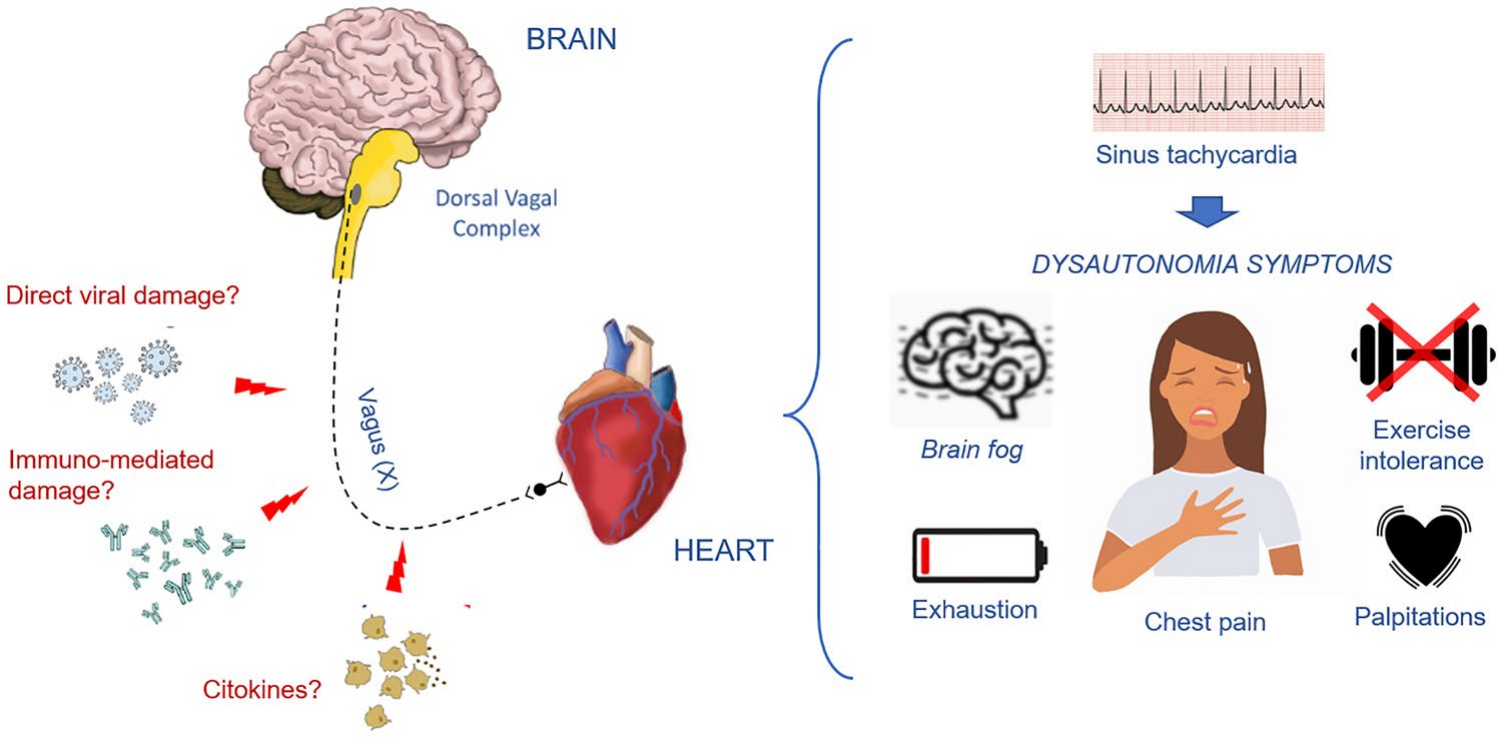
Illustration of the pathophysiological mechanisms underlying Post-COVID-19 syndrome.

To the best of our knowledge, this is the first prospective series of consecutive PCS patients in whom a comprehensive cardiovascular evaluation has been performed for the investigation of IST. We found that IST is prevalent among PCS patients (affecting 20% in our series), and this disorder was more common in young women without previous comorbidities and with mild SARS-CoV-2 infection. IST provides a plausible explanation for some of the prevalent symptoms of fatigue, impaired exercise capacity, and palpitations that characterize PCS and limit the affected individuals’ ability to carry out a normal life (Fig. 3). Finally, our results suggest a major role of the ANS in the pathophysiology of IST. This is supported by the 24-h ECG monitoring, as IST was accompanied by a decrease in most HRV parameters, predominantly during the daytime, and the most reduced components were those related to the cardiovagal tone (pNN50 and HF band). Accordingly, the loss of HRV is suggestive of a cardiac ANS imbalance with decreased parasympathetic activity and compensatory sympathetic activation. Hypoactivity of the parasympathetic tone could explain not only our findings of PCS-related IST, but also other prevalent symptoms in this setting, such as fatigue, gastrointestinal discomfort, headache, sore throat, neurocognitive disorder, and altered sleep structure (Central Illustration). Our findings are consistent with previous investigations suggesting that PCS could be a form of post-infectious dysautonomia.

Anxiety disorders, an acknowledged cause of sinus tachycardia, were not systematically evaluated in our patient population. However, the observed low HRV in our cohort and manifest physical limitations during the 6MWT makes anxiety-driven IST rather unlikely.

The mechanisms of IST, with or without previous viral infection, are poorly understood and investigated, but many of the postulated mechanisms include alterations in the nervous system: sympathovagal imbalance, beta-adrenergic receptor hypersensitivity, and brain stem dysregulation, among others. Considering this, it seems reasonable that the mechanisms leading to IST after SARS-CoV-2 infection are mixed, with injury of the ANS, which constantly regulates heart rate and vascular tone, playing an important role.

Some studies have shown that COVID-19 has significant cardiovascular involvement, but no previous research has focused on IST after SARS-CoV-2 infection. However, this is not the first time that IST has been described after coronavirus infection. In 2006, Yu et al. found that IST was the most common cardiovascular complication in a cohort of 121 patients with SARS. IST occurred in the absence of fever and was persistent in nearly 40% of patients during 3 weeks of follow-up. The authors observed that cardiovascular outcomes did not correlate with the occurrence of hypoxemia, admission to the intensive care unit, or analytical abnormalities^9^. Madjid et al. previously described a weak association between acute Severe Acute Respiratory Syndrome Coronavirus-1 (SARS-CoV-1) and acute Middle Respiratory Syndrome (MERS) and cardiovascular complications, such as arrhythmia and transient diastolic dysfunction. In addition, a review of 28 studies evaluating the long-term manifestations of SARS-CoV-1 and MERS observed that the most common symptoms were fatigue, dyspnea, and weakness, similar to PCS^10^.

On the one hand, post-infectious dysautonomia has previously been described in relation to other pathogens, including Chagas disease, human immunodeficiency virus (HIV), Epstein-Barr virus, and rabies virus^11,12^. Autonomic dysfunction is relatively common among HIV-infected patients, as inferred from a decrease in the HRV in the early stages of infection in many of these patients^13^. On the other hand, that patients with IST or POTS often report experiencing a previous trigger, such as a viral infection^14,15^. In previous observational studies, previous infectious illness was the precipitating event for IST in 5–10% of cases, and the reported pathogens were the influenza virus, Epstein-Barr virus, and herpes zoster, among others^16^. All of these studies mentioned ANS disruption. A number of mechanisms have been proposed to explain the occurrence of ANS dysfunction after a viral infection: denervation of the ANS, virus-dependent tissue damage due to persistent infection, and immune-mediated injury, among others. However, the prevalence and the mechanisms underlying the cardiovascular consequences of post-infectious dysautonomia are not clear and need to be investigated further.

As with other pathogens, there is convincing evidence that SARS-CoV-2 can damage the ANS. Previous studies have suggested a number of concurrent mechanisms, including direct brain invasion across the ethmoid bone or via the olfactory bulb during acute infection or blood dissemination of the virus and use of the ACE2 receptor for intracellular penetration. This receptor is also present on the glial cells and neurons. Several lines of evidence also support “indirect” mechanisms as the most important mechanisms involved in neurological injury, including vasculitis, thrombosis, and endothelial damage, along with exaggerated inflammation and immune responses^17–22^.

Furthermore, the evidence not only supports that SARS-CoV-2 can affect the nervous system during the acute phase, there is growing evidence in patients with orthostatic syndromes and syncope following SARS-CoV-2 infection that endorses a patho-physiological link between PCS and ANS dysfunction. A clear example of the capacity of the virus to alter the ANS is the so-called “silent hypoxia”, a characteristic sign of COVID-19. This condition has been associated with endothelial damage affecting the central and peripheral nervous receptors, altering respiratory control and dyspnea perception. Specifically, the injury has been postulated to occur in the vagal fibers, the glossopharyngeal afferents, and in the nucleus of the tractus solitarius, which are all key in respiratory and autonomic homeostasis^23,24^. In our study, most of the patients could not be evaluated for silent hypoxemia because arterial blood gases were not performed during the acute phase. Nevertheless, nearly all patients with silent hypoxemia are hospitalized at some point, as this condition leads to a critical diagnostic delay; in contrast to our study population of patients with mild disease who did not require hospital admission (therefore, assuming the absence of silent hypoxemia).

Finally, interference of angiotensin II synthesis by COVID-19 can be postulated as the last possible patho-physiological mechanism leading to dysautonomia. As discussed above, SARS-CoV-2 penetrates cells by attaching to the ACE2 receptor, influencing the synthesis of endogenous angiotensin II, a hormone that directly activates the SNS. Dysfunction of the renin–angiotensin–aldosterone system with compensatory activation of the SNS may also contribute to IST. However, our study was unable to demonstrate SNS participation in IST, and further investigations are needed to elucidate and characterize this patho-physiological aspect.

The reasons for the absolute predominance of this pathological phenomenon in young females, the concomitant high prevalence of environmental allergies, and the lack of correspondence with the severity of the index SARS-CoV-2 acute infection remain uncertain. Although IST and POTS are complex, heterogeneous syndromes with overlapping clinical manifestations and potential common mechanisms, it remains important to distinguish between these entities in order to provide the most appropriate treatment. The results of our study suggest that patients with PCS and IST may likely benefit from pharmacological treatment, such as beta-blockers, which blunt the sympathetic nervous system response. However, the pharmacological agent of choice, the timing of its administration, and the clinical response will warrant a separate investigation.

## Limitations

Assessment of ANS function is challenging and barely feasible in daily clinical practice. Standardized reference values extracted from healthy populations are frequently not available. For this reason, we performed the same tests in two gender- and age-controlled groups, one with matched disease stage and severity and one without previous infection. The small size of the control group is also a limitation, and the real incidence of the disease should be ascertained in larger population studies. Despite these limitations, we demonstrated significantly decreased parasympathetic tone among our PCS patient population.

Most of the patients included in this study did not require hospital admission during the acute phase of SARS-CoV-2 infection. Thus, laboratory parameters characterizing a presumable pro-inflammatory state and/or myocardial damage during the acute infection phase were not available.

## Conclusions

Inappropriate sinus tachycardia is a prevalent condition among PCS patients and should be incorporated as part of the myriad of multi-organ disorders comprising PCS. This disorder may at least partially explain the prevalent symptoms of palpitations, fatigue, and impaired exercise capacity observed in PCS patients. Cardiac ANS imbalance with decreased parasympathetic activity seems to be a plausible pathophysiological explanation for this phenomenon.
